# Stress hyperglycemia as a modifiable predictor of futile recanalization in patients undergoing mechanical thrombectomy for acute ischemic stroke

**DOI:** 10.3389/fneur.2023.1170215

**Published:** 2023-05-19

**Authors:** Giovanni Merlino, Sara Pez, Roberto Sartor, Fedra Kuris, Yan Tereshko, Lorenzo Nesi, Simone Lorenzut, Francesco Janes, Massimo Sponza, Vladimir Gavrilovic, Nicola Marotti, Andrea Pellegrin, Annarita Dapoto, Alessandro Vit, Alessandro Pauro, Gian Luigi Gigli, Mariarosaria Valente

**Affiliations:** ^1^Stroke Unit, Department of Head-Neck and Neuroscience, Udine University Hospital, Udine, Italy; ^2^Clinical Neurology, Udine University Hospital, Udine, Italy; ^3^Division of Vascular and Interventional Radiology, Udine University Hospital, Udine, Italy; ^4^Dipartimento di Area Medica (DAME), University of Udine, Udine, Italy

**Keywords:** stress hyperglycemia, mechanical thrombectomy, large vessel occlusion, acute ischemic stroke, futile recanalization

## Abstract

**Introduction:**

Mechanical thrombectomy (MT) is the first line treatment in acute ischemic stroke (AIS) due to large vessel occlusion (LVO). Approximately half of patients treated with MT does not have a favorable outcome 3 months after stroke. The aim of this study was to identify predictors of futile recanalization (FR) in patients with LVO treated with MT.

**Methods:**

A retrospective analysis of consecutive patients with acute ischemic stroke due to anterior circulation LVO who underwent MT. Patients with a TICI score of 2b or 3 were included. We distinguished two groups, FR and meaningful recanalization (MR), according to patients' disability three months after stroke (FR: mRS score > 2; MR: mRS score < 2).

**Results:**

We enrolled 238 patients (FR, *n* = 129, 54.2%; MR, *n* = 109, 45.8%). Age (OR 1.05, 95% CI 1.01–1.09, *p* = 0.012), female sex (OR 2.43, 95% CI 1.12–5.30, *p* = 0.025), stress hyperglycemia, as measured by the GAR index, (OR 1.17, 95% CI 1.06–1.29, *p* = 0.002), NIHSS at admission (OR 1.15, 95% CI 1.07–1.25, *p* = 0.001) and time from symptoms onset to MT (OR 1.01, 95% CI 1.00–1.01, *p* = 0.020) were independent predictors of FR. The AUC for the model combining age, female sex, GAR index, NIHSS at admission and time from symptoms onset to MT was 0.81 (95% CI 0.76–0.87; *p* < 0.001). The optimal GAR index cut-off score to predict FR was 17.9.

**Discussion:**

FR is common after MT. We recognized older age, female sex and baseline NIHSS as non-modifiable predictors of FR. On the other hand, time from symptoms onset to MT and stress hyperglycemia were modifiable pre- and post-MT factors, respectively. Any effort should be encouraged to reduce the impact of these modifiable predictors.

## Introduction

Mechanical thrombectomy (MT) is the first-line treatment in acute ischemic stroke (AIS) due to large vessel occlusion (LVO) (1). Despite recent trials showing that successful reperfusion, defined as a thrombolysis in cerebral infarction (TICI) score of 2b or 3, can be achieved in 80–90% of acute LVO patients treated with MT (2–4), approximately half of these patients does not have a favorable outcome, defined as a modified Rankin scale (mRS) of 0–2, 3 months after stroke (5, 6). This clinical condition, characterized by a successful recanalization without meaningful improvement in functional outcomes, is known as futile recanalization (FR).

Several mechanisms have been proposed to explain FR, namely, extensive tissue damage prior to MT, cerebral edema, the no-reflow phenomenon, and reperfusion injury that could cause FR (7). The knowledge of FR predictors might help stroke physicians improve their clinical management of AIS due to LVO. Differently from non-modifiable predictors, such as age and NIHSS score, that would suggest who will or will not benefit from MT, the identification of modifiable predictors of FR would change postoperative practice in order to reach long-term favorable outcomes. In particular, hyperglycemia, which is a common phenomenon in AIS patients, has been independently associated with adverse clinical outcomes (8). Hyperglycemia might favor FR by increasing lactic acidosis, reducing cerebral vasomotor reactivity and disrupting the blood-brain barrier, with an aggravation of cytotoxic edema, reduction of penumbra, impairment of collateral circulation, and increased risk of symptomatic intracranial hemorrhage (SICH) as consequences (9). Supporting the abovementioned hypotheses, we recently demonstrated that AIS patients affected by LVO with persistent hyperglycemia had a significantly increased risk of poor functional outcomes and mortality and hemorrhagic transformation after endovascular treatment. Since these detrimental effects were confirmed after excluding diabetic patients, we can hypothesize that stress hyperglycemia might also have a role in causing FR after MT (10).

Since a recent review encouraged collecting more evidence to reach a consensus on optimal postoperative management useful to minimize FR (7), we decided to perform this study with the aim of identifying modifiable, including stress hyperglycemia, and non-modifiable predictors of FR in AIS patients with LVO who had been treated with MT.

## Materials and methods

### Patient population

We conducted a single-center retrospective cohort observational study of consecutive patients affected by AIS due to LVO occlusion who were treated with MT at the Udine University Hospital from January 2015 to July 2022.

At our center, the eligibility criteria for MT are as follows: (1) presence of LVO in the anterior or posterior circulation as revealed by CT angiography (CTA), (2) onset of symptoms within 6 h, and (3) Alberta Stroke Program Early CT Score (ASPECTS) ≥6 on a direct CT scan (11). In contrast, patients with a life expectancy of < 6 months, severe medical conditions with signs of organ failure and platelet count < 55,000 mmc are not treated with MT at our center. Pre-stroke mRS does not represent an “absolute” exclusion criterion at our center. According to the international guidelines, alteplase is used to treat AIS patients showing onset of symptoms within 4.5 h (1).

Patients were included in the study if they achieved successful recanalization (TICI 2b or 3) after MT. In order to minimize the heterogeneity of the sample, we excluded patients who had posterior circulation occlusion.

Written informed consent was obtained from all patients or their representatives. The study conformed to the Declaration of Helsinki of the World Medical Association and was approved by the local ethics committee (Ref. No. CEUR-2020-Os-173).

### Data collection

We collected the following information: age, sex, vascular risk factors (previous transient ischemic attack or stroke, cardiovascular disease, atrial fibrillation, hypertension, diabetes mellitus, hypercholesterolemia, and active tobacco use), previous antithrombotic treatment, systolic blood pressure at admission, and laboratory findings, including stress hyperglycemia. Stress hyperglycemia was estimated by the GAR index (glucose-to-glycated hemoglobin ratio) that was calculated using the following formula: fasting plasma glucose (mg/dl)/HbA1c (%).

### Clinical assessment

According to the Trial of ORG 10172 in Acute Stroke Treatment (TOAST) criteria, ischemic strokes are classified into different subtypes based on etiology (12). Stroke severity was quantified at admission using the National Institutes of Health Stroke Scale (NIHSS) score. The degree of previous functional disability was calculated at admission, based on pre-stroke disability, and 3 months after stroke using the modified Rankin scale (mRS). The mRS score after discharge was recorded during the patients' routine clinical visits or through telephone interviews with patients or their immediate caregivers. ASPECTS was used for grading early ischemic changes within the MCA territory on a native scan (11). Based on the ECASS III protocol, the presence of symptomatic intracranial hemorrhage (SICH) was defined as any hemorrhage with neurological deterioration, as indicated by an NIHSS score that was higher by ≥4 points than the value at the baseline or the lowest value in the first 7 days or any hemorrhage leading to death (13). All MT patients received a follow-up CT scan ~24 h after recanalization therapy or sooner if clinical deterioration was observed.

### Thrombectomy procedure

All thrombectomy procedures were performed by experienced interventional radiologists (M.S., V.G., N.M., A.P., A.D., A.V., and A.P.). At our center, a direct aspiration first-pass technique is used as first-line therapy in AIS patients with LVO. In case of failure, stent retriever devices in conjunction with distal aspiration through intermediate catheters are used. In cases of stent retriever thrombectomy failures, a permanent stent is deployed in the occluded segment. In AIS patients, tandem occlusion emergent stenting of the extracranial internal carotid artery is performed at the discretion of the treating physician. Patients received local or general anesthesia with endotracheal intubation at the discretion of the anesthesiologists. The angiographic result was assessed on the final digital subtraction angiography image series and was classified according to the original version of the TICI score (14). TICI 2b was defined as “restoration of more than two-thirds of the target downstream territory” and TICI 3 as “complete reperfusion.”

The following information was collected: site of occlusion, type of device used for the MT procedure, number of retrieval attempts, presence of secondary embolization, time from onset of symptoms to groin puncture, time from hospital arrival to groin puncture (door-to-groin time), and procedure duration. In addition, if patients received alteplase, we collected information on the time from onset of symptoms and from hospital arrival to alteplase administration (door-to-needle time).

### Statistical analysis

Patients included in the study were divided into two groups, FR and meaningful recanalization (MR), according to their disability status 3 months after stroke. The FR group included all patients with a 3 month mRS score > 2, whereas patients were allocated to the MR group if they had a 3 month mRS score ≤ 2. For patients with a pre-stroke mRS > 2, MR was defined as a return to the pre-stroke mRS 3 months after AIS. If it did not occur, patients were allocated to the FR group.

Statistical comparisons were performed using the chi-square test or Fisher's exact test, when appropriate, for categorical variables. Differences between the two groups were assessed by means of the Student *t*-test for independent samples when variables had a normal distribution and by the Mann–Whitney U-test when variables had an abnormal distribution. The Kolmogorov–Smirnov test with Lilliefors significant correction was performed to test the normality of the variables.

A binary logistic regression model was used for detecting independent predictors of FR. This model was adjusted for all the variables with a probability value of < 0.05 in univariate analysis. Multicollinearity was assessed by calculating variance inflation factors (VIFs) for variables included in the model, and a significant multicollinearity was defined as a VIF >10.

The diagnostic values of factors, solely or in combination, to predict FR were tested with area under the receiver operating characteristic curves (AUC-ROC). The Youden index was performed to identify the optimal cutoff score for the GAR index. The Youden index seeks to identify the point on the ROC curve that maximizes the equation “J = sensitivity + specificity−1.” A test's cutoff score with the highest Youden index optimally balances sensitivity and specificity (15).

Data are displayed in tables as mean and standard deviation unless otherwise specified.

All probability values were two-tailed. Statistical significance was set at a *p*-value of < 0.05. Statistical analysis was carried out using IBM SPSS Statistics for Windows, version 22.0 (IBM Corp., Armonk, NY, USA).

## Results

**Figure 1:**
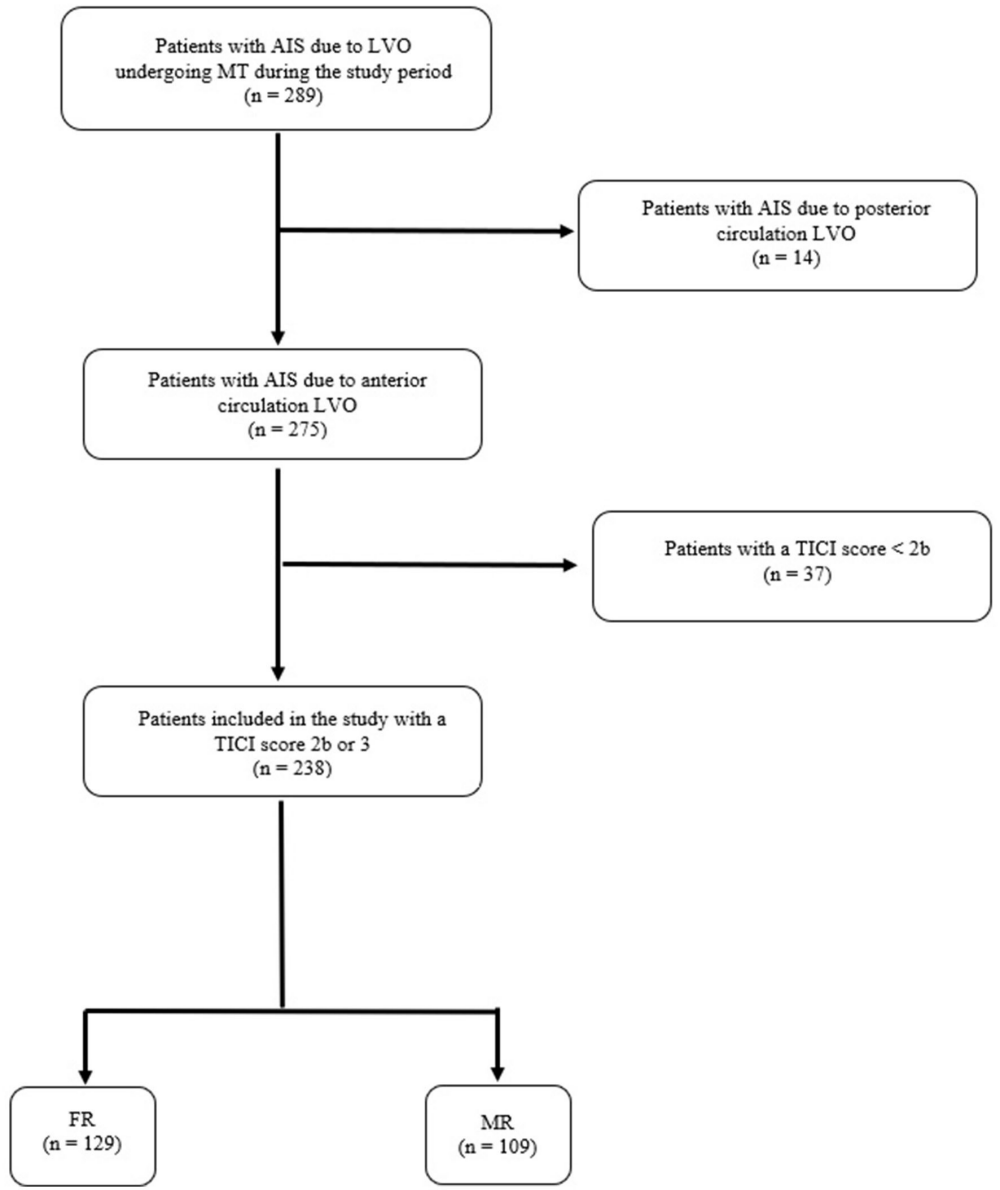
Flow diagram of the study. AIS, acute ischemic stroke; LVO, large vessel occlusion; TICI score, thrombolysis in cerebral infarction score; FR, futile recanalization; MR, meaningful recanalization.

During the study period, 289 patients were treated with MT for AIS due to LVO occlusion. Of these, 14 patients were excluded because of AIS due to posterior circulation LVO. Of the remaining 275 patients with AIS due to anterior circulation LVO, 37 patients (13.5%) were excluded because they did not achieve a TICI score of 2 or 3 after MT. Thus, 238 subjects (86.5%) were included in the study and distinguished into two groups, FR (*n* = 129, 54.2%) and MR (*n* = 109, 45.8%). These data are summarized in the flow diagram of the study (Figure 1).

The general characteristics of patients included in the two groups are presented in Table 1. Patients with FR were older and more frequently female than those in the MR group. Moreover, the presence of FR was significantly associated with a higher score of NIHSS and a lower score of ASPECTS. Regarding laboratory findings, hemoglobin, INR, C-reactive protein, and glycemic profile were significantly different between the two groups.

**Table 1:** General characteristics of the patients included in the futile recanalization and meaningful recanalization groups.

	**FR (*n* = 129)**	**MR (*n* = 109)**	***p***
**Demographic data**			
Age, years^*^	79 (71–83)	72 (63.2–78)	**0.001**
Females, *n* (%)	76 (58.9)	50 (45.9)	**0.045**
**Vascular risk factors**			
Previous transient ischemic attack/stroke, *n* (%)	12 (9.3)	13 (11.9)	0.511
Cardiovascular disease, *n* (%)	16 (12.4)	19 (17.4)	0.275
Atrial fibrillation, *n* (%)	41 (31.8)	24 (22)	0.092
Hypertension, *n* (%)	94 (72.9)	67 (61.5)	0.061
Diabetes mellitus, *n* (%)	19 (14.7)	14 (12.8)	0.675
Hypercholesterolemia, *n* (%)	34 (26.4)	28 (25.7)	0.907
Current smoking, *n* (%)	15 (12.4)	21 (20.2)	0.112
**Previous antithrombotic treatment**			0.488
Antiplatelets, *n* (%)	32 (24.8)	27 (24.8)	
Anticoagulants, *n* (%)	25 (19.4)	15 (13.8)	
Systolic blood pressure at admission, mmHg	152.6 ± 24.6	149.6 ± 21.6	0.387
**Laboratory findings**			
Hb, g/dl^*^	12.2 (11–13.9)	12 (13–14)	0.072
Platelets, 10^3^/mmc^*^	188 (150–232.7)	202 (162–251)	0.076
aPTT ratio	1.03 ± 0.22	0.99 ± 0.16	0.145
INR	1.11 ± 0.21	1.05 ± 0.17	**0.041**
Creatinine, mg/dl^*^	0.92 (0.73–1.12)	0.84 (0.76–1.06)	0.297
C-reactive protein, mg/l^*^	10.05 (3.86–23.26)	6 (2.1–12.45)	**0.002**
Fasting glucose, mg/dl^*^	113 (98.8–147)	98 (89–112)	**0.001**
HbA1c values, %^*^	5.9 (5.5–6.3)	5.7 (5.4–6)	**0.030**
GAR index^*^	19.6 (17.4–23.2)	17.6 (15.6–19.8)	**0.001**
Total cholesterol, mg/dl	160.7 ± 35.7	166.2 ± 38.8	0.271
HDL cholesterol, mg/dl^*^	51 (39.5–59)	51 (42–62)	0.549
LDL cholesterol, mg/dl	90.3 ± 30.9	94.6 ± 33.6	0.323
Triglycerides, mg/dl^*^	87.5 (71.2–133)	93 (69–131)	0.958
**Previous antithrombotic treatment**			0.488
Antiplatelets, *n* (%)	32 (24.8)	27 (24.8)	
Anticoagulants, *n* (%)	25 (19.4)	15 (13.8)	
**Stroke subtypes based on TOAST classification**			0.613
Large arterial atherosclerosis, *n* (%)	13 (10.1)	17 (15.5)	
Cardioembolism*, n* (%)	76 (58.9)	58 (53.2)	
Other determined etiology, *n* (%)	9 (7)	7 (6.4)	
Undetermined etiology, *n* (%)	31 (24)	27 (24.8)	
**Baseline clinical characteristics**			
NIHSS score at admission, median (IQR)	18 (16–21)	14 (10–18)	**0.001**
Pre-stroke mRS 0–2, *n* (%)	118 (91.5)	105 (96.3)	0.124
Baseline ASPECTS, median (range)	10 (6–10)	10 (7–10)	**0.001**
Presence of SICH, *n* (%)	17 (13.2)	3 (2.9)	**0.004**

FR, futile recanalization; MR, meaningful recanalization; Hb, hemoglobin; aPTT, activated partial thromboplastin time; INR, international normalized ratio; HbA1C, glycated hemoglobin; GAR, glucose-to-glycated hemoglobin ratio; HDL, high-density lipoprotein; LDL, low-density lipoprotein; TOAST, Trial of ORG 10172 in Acute Stroke Treatment; NIHSS, National Institute of Health Stroke Scale; mRS, modified Rankin scale; ASPECTS, Alberta Stroke Program Early CT Score; SICH, symptomatic intracranial hemorrhage. Data are presented as mean and standard deviation for normally distributed continuous variables. Non-normally distributed continuous variables are displayed as median and interquartile range and are identified by an asterisk (^*^). Bold values denote statistical significance at a p-value of < 0.05.

Patients with FR were more frequently affected by tandem occlusions and treated with a combination of thromboaspiration plus stent retriever than subjects in the MR group. In addition, time from symptom onset to MT and duration of the procedure were significantly longer in FR patients than in MR ones. Finally, the use of alteplase was significantly associated with MR (see Table 2).

**Table 2:** Information on the thrombectomy procedure of the patients included in the futile recanalization and meaningful recanalization groups.

	**FR (*n* = 129)**	**MR (*n* = 109)**	***p***
**Site of occlusion**			**0.024**
MCA, *n* (%)	96 (74.4)	94 (86.2)	
Tandem, *n* (%)	33 (25.6)	15 (13.8)	
**Type of device use for MT**			**0.029**
Thromboaspiration, *n* (%)	52 (40.3)	64 (58.7)	
Stent retriever, *n* (%)	5 (3.9)	4 (3.7)	
Thromboaspiration plus stent retriever, *n* (%)	58 (45)	30 (27.5)	
Permanent stenting, *n* (%)	14 (10.9)	11 (10.1)	
**Other information on recanalization therapy**			
Number of retrieval attempts >3, *n* (%)	44 (34.1)	25 (22.9)	0.058
Secondary embolization, *n* (%)	7 (5.4)	8 (7.3)	0.545
Time from symptoms onset to MT, min^*^	210 (170–270)	195 (150–235)	**0.022**
Door-to-groin time, min^*^	116.5 (85–154.2)	110 (85–135.5)	0.157
Procedure length, min^*^	70 (45–95)	60 (40–82.5)	**0.028**
Alteplase use prior to MT, *n* (%)	67 (51.9)	71 (65.1)	**0.040**
Time from symptoms onset to alteplase, min^*^	150 (110–171.5)	136 (105–170)	0.496
Door-to-needle time, min^*^	60 (35.5–78)	55 (41–70)	0.605
**TICI score after MT**			**0.152**
TICI 2b, *n* (%)	39 (61.9)	24 (38.1)	
TICI 3, *n* (%)	90 (51.4)	85 (48.6)	

FR, futile recanalization; MR, meaningful recanalization; MCA, middle cerebral artery; MT, mechanical thrombectomy; TICI score, thrombolysis in cerebral infarction score. Data are presented as mean and standard deviation for normally distributed continuous variables. Non-normally distributed continuous variables are displayed as median and interquartile range and are identified by an asterisk (^*^). Bold values denote statistical significance at a p-value of < 0.05.

Our binary logistic regression model showed that age, female sex, GAR index, NIHSS at admission, and time from symptom onset to MT were significantly associated with clinically ineffective reperfusion after MT (see Table 3).

**Table 3:** Odds ratio (95% confidence intervals) for futile recanalization.

**Futile recanalization**	**OR**	**95% CI**	***p***
Age	1.05	1.01–1.09	**0.012**
Females	2.43	1.12–5.30	**0.025**
INR	2.00	0.35–11.48	0.435
C-reactive protein	1.01	0.99–1.03	0.131
GAR index	1.17	1.06–1.29	**0.002**
NIHSS at admission	1.15	1.07–1.25	**0.001**
ASPECTS	0.65	0.42–1.01	0.057
SICH	3.63	0.62–21.17	0.152
**Site of occlusion**			
*MCA*	1		
*Tandem*	1.62	0.62–4.27	0.326
**Type of device**			
*Thromboaspiration*	1		
*Stent retriever*	0.43	0.04–4.99	0.500
*Thromboaspiration + stent retriever*	1.71	0.73–3.98	0.215
*Permanent stenting*	0.70	0.16–3.09	0.637
Time from symptoms onset to MT	1.01	1.00–1.01	**0.020**
Procedure length	1.01	0.99–1.02	0.242
Alteplase use prior to MT	0.80	0.36–1.82	0.601

INR, international normalized ratio; GAR, glucose-to-glycated hemoglobin ratio; NIHSS, National Institute of Health Stroke Scale; ASPECTS, Alberta Stroke Program Early CT Score; SICH, symptomatic intracranial hemorrhage; MCA, middle cerebral artery; MT, mechanical thrombectomy. Bold values denote statistical significance at a p-value of < 0.05.

**Figure 2:**
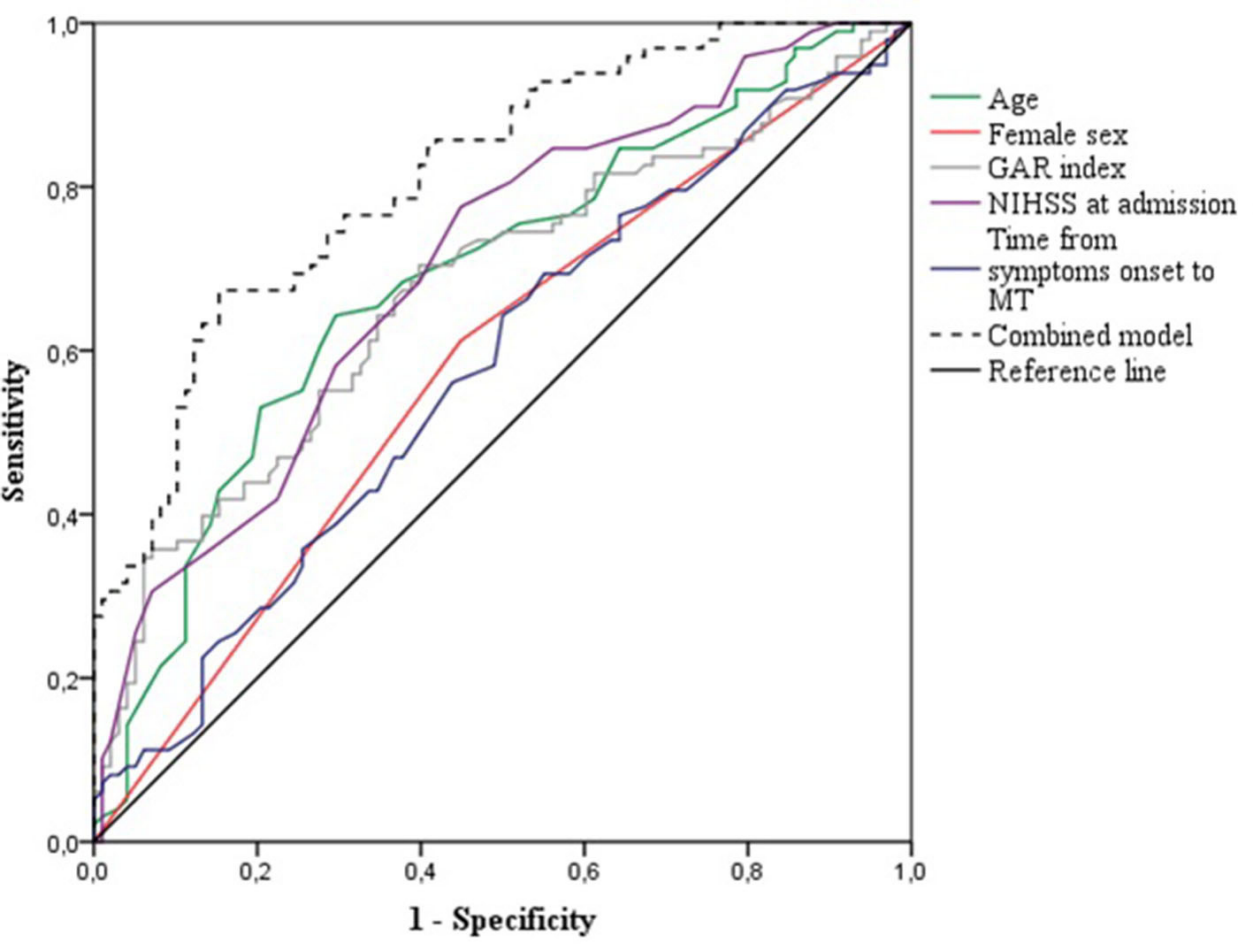
Receiver operating characteristics curves for the prediction of futile recanalization after mechanical thrombectomy. GAR, glucose-to-glycated hemoglobin ratio; NIHSS, National Institute of Health Stroke Scale; MT, mechanical thrombectomy.

Using the ROC curves from the logistic regression analysis, we identified the predictive accuracy of age, female sex, GAR index, NIHSS at admission, and time from symptoms onset to MT for predicting FR (see Figure 2). The AUC for age, female sex, GAR index, NIHSS at admission, and time from symptoms onset to MT were 0.69 (95% CI 0.61–0.76, *p* < 0.001), 0.58 (95% CI 0.50–0.64, *p* = 0.048), 0.67 (95% CI 0.60–0.75, *p* < 0.001), 0.70 (95% CI 0.63–0.77, *p* < 0.001), and 0.58 (95% CI 0.50–0.66, *p* = 0.064), respectively. The model combining age, female sex, GAR index, NIHSS at admission, and time from symptom onset to MT had the highest AUC (0.81, 95% CI 0.76–0.87, *p* < 0.001). The optimal GAR index cutoff score to predict FR was 17.9, with a Youden index of 0.31, a sensitivity of 0.70, and a specificity of 0.61.

## Discussion

Almost half (54%) of our patients with successful recanalization after MT did not achieve favorable outcomes 3 months after AIS due to anterior circulation LVO. This result is in line with previous studies reporting an FR rate comprised between 49.6 and 59.8% (16). We recognized five independent predictors of FR. Indeed, older age, female sex, stress hyperglycemia, baseline NIHSS score, and time from stroke onset to the procedure were significantly associated with clinically ineffective reperfusion after controlling for several confounders in the binary logistic model.

MT is the standard of care for patients presenting with AIS due to LVO (1). Despite recent advances in devices allowing a high recanalization rate of over 70%, to date, functional independence in AIS patients is only approximately 50% (2, 17). Thus, the interest of the international scientific community shifted toward potential causes and mechanisms of FR (7). Although several non-modifiable and modifiable prior to recanalization factors have been identified as predictors of FR, Nie et al. (7) urged to perform new studies for defining some specific measures of immediate post-MT care in order to achieve a favorable outcome. Our analysis demonstrated that stress hyperglycemia, a modifiable post-MT factor, is strictly and independently associated with FR. Moreover, a GAR index of 17.9 seems to be the optimal cutoff score able to distinguish between patients with favorable or unfavorable outcomes 3 months after stroke. Although previous studies have shown that stress hyperglycemia, measured by the GAR index, is able to impair outcomes of AIS patients treated with MT (18), no research investigated its role as a predictor of FR.

Several mechanisms might mediate our association between stress hyperglycemia and FR. It is known that hyperglycemia leads to a hypercoagulable state due to elevated plasminogen activator inhibitor type 1 (PAI-1) and decreased plasminogen activator (t-PA) activity levels (19, 20). In addition, hyperglycemia is able to cause deleterious effects on cerebral microvasculature, which is where the no-reflow phenomenon occurs. An animal model experiment by Fabian and Kant demonstrated that high glycemic levels induce a state of “functional uncoupling” in the cerebral microvasculature of the peri-infarct region, generating superoxide radical in lieu of nitric oxide. Thus, therapeutic interventions on stress hyperglycemia might correct or ameliorate these changes reducing the cerebral injury after recanalization (21). The optimal treatment for stress hyperglycemia remains an unsettled question. Previous trials did not show any significant benefit of tight glycemic control on functional outcomes or in survival (22). Since GAR seems to be a strong predictor of poor prognosis, we propose that stratified glycemic targets based on GAR values rather than the absolute glucose value may be applied to the management of stress hyperglycemia in future studies.

Although a recent meta-analysis by Deng et al. (16) suggests that serum glucose at admission increases the risk of FR (mean difference 0.59, 95% CI 0.37–0.81, *p* < 0.00001), results coming from every single study included in the meta-analysis do not support this conclusion (23–27). In 2013, Singer et al. (23) investigated predictors of FR in a sample of 223 patients treated with endovascular therapy for anterior LVO. The authors observed that median levels of baseline serum glucose were significantly higher among subjects with FR than in those included in the successful recanalization group (124 vs. 113 mg/dl, *p* < 0.05). However, since the authors did not perform multivariate analysis, these preliminary results cannot be considered conclusive (23). A further study by Hussein et al. (24) reported increased mean levels of serum glucose at admission (7.6 vs. 6.7 mmol/l, *p* = 0.039) in the group of patients with FR, but this association was lost after performing a stepwise linear regression analysis. Similar results have been reported by Xu et al. (25) that investigated FR in the Chinese population. Indeed, patients with FR had a higher baseline serum glucose than those with MR, but this association was not confirmed after multivariate analysis. Differently, Zang et al. (26) and Mechtouff et al. (27) reported that admission glucose levels were superimposable between FR and MR patients.

In our sample, we observed an odds ratio of 1.05 (95% CI 1.01–1.09) for FR for each year of age increase. Older age is a recognized predictor for futile recanalization after MT (16). Singer et al. (23) reported that the proportion of patients with futile recanalization increased from 29% (18–53 years) to 34% (54–67 years) and 40% (68–76 years), peaking at 53% in patients within the highest age quartile (77–94 years). This significant association between older age and FR has been recently confirmed by the meta-analysis of Deng et al. (16) (mean difference 5.91, 95% CI 4.16–7.46, *p* < 0.00001). Pre-existing physical disabilities, dementia, infectious and non-infectious complications, high rate of cardioembolic strokes, and less rehabilitation potential could explain this relationship (16). However, strong data support the beneficial impact of MT in patients older than 80 years (5, 28). Thus, no upper age limit should be settled for MT.

Our females had more than twice the risk of FR than male patients. Similarly, Hussein et al. (24) found that most men in whom successful recanalization was achieved had favorable outcomes (56.5%) unlike women (38.7%). Stroke burden is greater in women than in men. Women have less favorable outcomes after stroke with more physical impairments and limitations in activities of daily living (29). Moreover, a higher case fatality in women was shown in the International Stroke Trial (30). Sex differences in stroke outcomes might be related to sex steroid hormones, particularly estrogen. Since estrogen promotes dilation and blood flow, improves cerebrovascular reactivity, and has anti-inflammatory effects (29), post-menopausal women, not receiving estrogen replacement therapy, might have a poor recovery after AIS even though they underwent MT.

Our patients with FR had a worse neurological status, as measured by the NIHSS, at hospital admission than MR ones. This association was confirmed after controlling confounders. Although a higher baseline NIHSS score represents a recognized predictor of FR (16), it should not preclude MT in subjects with severe stroke. Indeed, Lee et al. (31) demonstrated that FR was associated with stroke severity, but at the same time, the therapeutic benefit of MT increased with increasing stroke severity (*p* for interaction < 0.001): 0.1% in the NIHSS score of ≤ 5, 18.6% in the NIHSS score of 6–10, 28.7% in the NIHSS score 11–20, and 34.3% in the NIHSS score of >20.

The odds of better disability outcomes at 90 days with endovascular therapy decline with a longer time from symptom onset to arterial puncture. Meta-analysis by the Hermes collaboration found that each hour delay to reperfusion was associated with a less favorable degree of disability (OR 0.84, 95% CI 0.76 to 0.93) (32). Hussein reported a significantly shorter time from AIS onset to endovascular treatment among MR patients and in those with unsuccessful recanalization (239.2 ± 47.7 vs. 265.8 ± 48.3, *p* = 0.042) (24). We confirmed that this modifiable pre-MT factor is independently associated with FR. Hence, time from symptom onset to MT should be shortened as much as possible for preventing unsuccessful recanalization. Our study stimulates us to achieve consensus on a future recommendation to bypass the primary stroke center and to go straight to the endovascular-capable hospital in patients with suspected large vessel occlusion. Moreover, the recently proposed one-stop stroke management platform, that combines computed tomography, magnetic resonance imaging, and digital subtraction angiography equipment in one space, seems to reduce in-hospital delays in the thrombectomy treatment of patients with stroke compared to traditional workflow (33).

Our study is subject to some limitations. First, the retrospective design of the study may produce systematic error and bias. Second, the observed associations are not proofs of causality; thus, our results should be considered as hypothesis generating. Third, the relatively small sample size may have limited the statistical power; thus, other significant differences between the two groups could not be detected. Finally, the definition of meaningful and futile recanalization for patients with pre-stroke mRS > 2 was arbitrary.

## Conclusion

Despite recent advances in devices and technologies, this study confirms that FR is common after MT. We recognized older age, female sex, and baseline NIHSS as non-modifiable predictors of FR. Since time from symptom onset to endovascular treatment was a modifiable pre-MT factor, we strongly support pre-hospital and in-hospital strategies able to reduce delays in the treatment of patients with stroke. Regarding stress hyperglycemia, a modifiable post-MT factor, this is the first study recognizing this variable as an independent predictor of FR. Thus, further studies are needed to confirm this relationship. Until then, we suggest paying particular attention to patients who underwent MT for anterior circulation LVO showing a GAR index of more than 17.9.

## Data availability statement

The raw data supporting the conclusions of this article will be made available by the authors, without undue reservation.

## Ethics statement

The studies involving human participants were reviewed and approved by Comitato Etico Locale (Ref. No. CEUR-2020-Os-173). The patients/participants provided their written informed consent to participate in this study.

## Author contributions

GM: conceptualization, methodology, formal analysis, data curation, writing—original draft preparation, and writing—review and editing. SP, RS, FK, YT, SL, and FJ: software and investigation. GG and MV: validation. MS, VG, NM, APe, AD, AV, and APa: resources. GG: visualization. MV: supervision. All authors contributed to the article and approved the submitted version.
